# Evaluation of glucose-6-phosphate dehydrogenase serum level in patients with multiple sclerosis and neuromyelitis optica

**Published:** 2019-10-07

**Authors:** Niloofar Chitsaz, Leila Dehghani, Amir Safi, Nazgol Esmalian-Afyouni, Vahid Shaygannejad, Majid Rezvani, Karim Sohrabi, Kaykhosro Moridi, Milad Moayednia

**Affiliations:** 1 Isfahan Neurosciences Research Center, Isfahan University of Medical Sciences, Isfahan, Iran; 2 Department of Tissue Engineering and Regenerative Medicine, School of Advanced Technologies in Medicine, Shahid Beheshti University of Medical Sciences, Tehran, Iran; 3 Department of Neurosurgery, School of Medicine, Isfahan University of Medical Sciences, Isfahan, Iran; 4 Department of Biology, School of Advanced Sciences and Technology, Pharmaceutical Sciences Branch, Islamic Azad University, Tehran, Iran

**Keywords:** Multiple Sclerosis, Neuromyelitis Optica, Vitamin D, Oxidative Stress, Glucosephosphate Dehydrogenase

## Abstract

**Background:** Multiple sclerosis (MS) and neuromyelitis optica (NMO) are both demyelinating disorders and oxidative stress is suggested to have a role in their pathogenesis. Glucose-6-phosphate dehydrogenase (G6PD) produces nicotinamide adenine dinucleotide phosphate (NADPH) via the pentose phosphate pathway. NADPH is not only involved in the synthesis of fatty acids necessary for myelination, but also it is involved in the defense against oxidative stress. Prescribing supplementary vitamin D as a part of the MS treatment plan can increase G6PD gene expression. The aim of this study was to determine the serum level of G6PD in patients with MS and NMO and its relationship with vitamin D, since it is yet to be explored thoroughly.

**Methods:** In this case-control study, subjects were divided into three experimental and control groups. The experimental groups comprised 50 patients with relapsing-remitting MS (RRMS) who had a history of vitamin D consumption, 50 newly-diagnosed MS patients, and 50 patients with NMO. Control group included 65 healthy individuals. Serum level of G6PD was measured and compared among these groups.

**Results:** No significant difference was seen between the G6PD level in patients with MS and NMO, but it should be noted that this level was significantly lower than the healthy group. G6PD serum level was significantly higher in patients with MS who had previously consumed supplementary vitamin D compared to those who had not.

**Conclusion:** G6PD deficiency is observed in patients with MS and NMO. Also, supplementary vitamin D may induce favorable results on the G6PD level.

## Introduction

Multiple sclerosis (MS) and neuromyelitis optica (NMO), also called Devic's disease, are autoimmune-mediated diseases of the central nervous system (CNS).^1^^,^^2^ The lesions of MS include the optic nerves, spinal cord, brainstem, cerebellum, and juxtacortical and periventricular white matter regions; while in NMO, optic nerves and the spinal cord are preferentially involved.^3^ As these two diseases have many overlapping symptoms, NMO has long been considered as a subtype of MS.^4^ However, since the discovery of the disease-specific autoantibody to NMO (AQP4-Ab), it is confirmed that these two diseases have distinct features in their epidemiology, serology, pathology, response to treatment, and prognosis.^5^ It is suspected that the oxidative stress is a key mechanism driving demyelination and neurodegeneration in these diseases.^6^ In general, oxidative stress occurs by an imbalance between production and accumulation of reactive oxygen species (ROS) like superoxide (O_2_^-^), hydrogen peroxide (H_2_O_2_), or hydroxyl radicals (OH) and the inability to detoxify them or to repair the resulting damage.^7^ Protection against oxidative damage largely relies on the reductive power of nicotinamide adenine dinucleotide phosphate (NADPH) generated by glucose-6-phosphate dehydrogenase (G6PD) in the pentose phosphate pathway.^8^ Indeed, G6PD deficiency might be considered as an important risk factor in the development of some neurodegenerative diseases.^9^ Also, the need for NADPH for the synthesis of fatty acids, which are myelin precursors, is crucially dependent on G6PD activity in oligodendroglia.^10^ This enzyme’s activity is high in myelinated fibers and varies relative to the amount of neuron’s myelination.^11^ Hence, demyelination in MS and NMO diseases can be attributed to G6PD deficiency. Vitamin D, used in MS treatment, can increase the activity and the production of G6PD.^12^^-^^14^ This can possibly explain the protective impact of vitamin D against oxidative stress.^15^ Considering G6PD’s protective role against oxidative damage as well as its role in myelin production, its deficiency may exist in MS and NMO. Therefore, the present study aimed at evaluation of G6PD level and the effect of vitamin D on it in patients who suffered from MS and NMO.

## Materials and Methods

In this case-control study, a total of 215 blood samples were collected from subjects attended to the neurology ward, Kashani Hospital, Isfahan, Iran, during 2015-2016. The subjects of the study included 50 patients with relapsing-remitting MS (RRMS) who previously had consumed vitamin D as their treatment plan (received 50000 units of vitamin D every two weeks in the 6 months as a supplement), 50 newly-diagnosed MS patients who had not used vitamin D3 which were selected based on 2010 revised McDonald criteria,^16^ 50 patients who suffered from NMO based on revised diagnostic criteria for NMO by Wingerchuk et al.,^17^ and 65 healthy controls. All of the participants were 16-55 years old including both genders who filled and signed the consent form. The study was also approved by the Ethics Committee of Isfahan University of Medical Sciences, Isfahan.

Serum level of G6PD was measured and compared among these groups. After collecting of blood samples, serum samples were isolated and frozen at -70 °C until analysis. In order to measure G6PD level, Human G6PD enzyme-linked immunosorbent assay (ELISA) kit (EASTBIOPHARM CO., LTD., China) was used. 

The analysis of the data was done using SPSS software (version 22, IBM Corporation, Armonk, NY, USA). The normalization of data was confirmed with Kolmogorov-Smirnov test. Patients’ characteristics were descriptively analyzed. The difference of G6PD as dependent variable among groups was examined using one-way ANOVA test. Moreover, the relevance of the G6PD level and the incidence of diseases was examined by the logistic regression. A P-value < 0.05 was considered statistically significant and the data were represented as mean ± standard deviation (SD).

## Results

Demographic data of patients with MS and NMO is summarized in table 1. The sex and age of individuals were analyzed among groups and none showed a significant difference (P > 0.050).

According to the results, no significant difference was seen between G6PD level in patients with MS and NMO. G6PD level in patients with NMO was significantly lower than that in the healthy group (17.685 ± 2.682 vs. 82.185 ± 7.867) (P < 0.001). Similarly, G6PD level in patients with MS was lower than that in the healthy group (16.465 ± 1.290 vs. 82.185 ± 7.867) (P < 0.001). 

**Table 1:** Demographic characteristics of healthy and patients groups

**Characteristics**		**Healthy ** **(n = 65)**	**RRMS ** **(n = 50)**	**Newly-diagnosed ** **MS (n = 50)**	**NMO ** **(n = 50)**	**P**
Sex [n (%)]	Female	40 (62.5)	36 (71.5)	34 (67.5)	32 (65.0)	0.803
	Male	25 (37.5)	14 (29.5)	16 (32.5)	18 (35.0)	
Age (year) (mean ± SD)		34.83 ± 10.07	35.80 ± 10.11	33.60 ± 9.91	34.77 ± 10.26	0.687

MS: Multiple sclerosis; RRMS: Relapsing-remitting multiple sclerosis; NMO: Neuromyelitis optica; SD: Standard deviation

**Figure 1:**
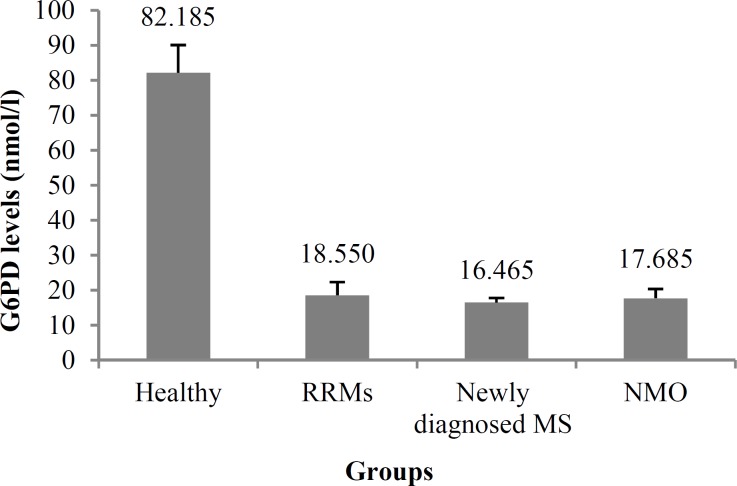
Glucose-6-phosphate dehydrogenase (G6PD) serum level of control and patients groups

G6PD level in patients with MS with consumption of vitamin D history was higher compared to patients with MS not treated by vitamin D (18.550 ± 2.762 vs. 16.465 ± 1.290) (P < 0.05), but still well below the level in the healthy group (18.550 ± 2.762 vs. 82.185 ± 7.867) (P < 0.001) (Figure 1).

Logistic regression analysis showed a highly significant effect of the G6PD level on being healthy or not. The G6PD level in all patients was lower than healthy persons [odds ratio (OR): 83, P < 0.001].

## Discussion

In the present study, G6PD deficiency was observed in patients with MS and NMO. Although the pathogenesis of MS is not completely understood, several studies suggest that ROS, which can be neutralized by G6PD, contribute to the formation and persistence of MS lesions.^18^ Genetic studies conducted by Fischer et al. indicated the prominent role of oxidative damage in demyelination and tissue damage in MS.^19^ Also, for NMO, despite the hypothesis claiming the relationship between ROS and oxidative damage and pathogenic aspects of this disease, it has been rarely investigated.^20^ Increased production of serum markers of oxidative stress, such as malondialdehyde (MDA), peroxidation potential, catalase, and hydroperoxides in general, in patients with NMO was reported in Penton-Rol et al.’s study.^21^ Peng et al. studied the relationship between NMO and the antioxidant status of uric acid, bilirubin, and albumin. They suggested that patients with NMO and low antioxidant status were not able to handle the toxicity of free radicals that lead to inflammation, neurodegeneration, and demyelination.^22^ Considering the critical role of G6PD in neutralizing oxidants, G6PD level in different areas of the CNS has been studied in different neurodegenerative diseases including Alzheimer's disease (AD) and Parkinson's disease (PD).^23^^,^^24^ However, no report on the level of this enzyme in patients with MS and NMO is available.

Considering the uncertain pathogenesis of MS, there are various treatment plans suggested for patients with MS, among them is vitamin D. 1,25-dihydroxyvitamin D3 (1,25-VD) has general neuroprotective and anti-inflammatory effects.^25^ Anti-oxidative effects of vitamin D3 may be attributed to the increased expression of G6PD. Genetic studies indicate that G6PD gene is the primary target gene for 1,25-VD.^12^ Vitamin D also increases G6PD activity.^14^ The findings of the current study, while supporting previous reports, suggest that vitamin D increases G6PD serum levels.

## Conclusion

Our results showed that G6PD serum level in both MS and NMO patients was low. In other words, whatever the amount of G6PD is lower, the probability of being sick is more. This can relatively explain the oxidative stress and demyelination observed in pathogenesis aspects of these two diseases. Neuroprotective role of G6PD can be considered in designing therapeutic plans for patients. Moreover, considering the increasing effect of vitamin D on G6PD serum level, new therapeutic options may be available for patients suffering from G6PD enzyme deficiency. Nonetheless, prescribing vitamin D supplements needs to be researched more in future.

## Notes:

**How to cite this article:** Chitsaz N, Dehghani L, Safi A, Esmalian-Afyouni N, Shaygannejad V, Rezvani M, et al. Evaluation of glucose-6-phosphate dehydrogenase serum level in patients with multiple sclerosis and neuromyelitis optica. Iran J Neurol 2019; 18(4): 150-3.

## References

[1] Dobson R, Giovannoni G (2019). Multiple sclerosis - a review. Eur J Neurol.

[2] Bruscolini A, Sacchetti M, La Cava M, Gharbiya M, Ralli M, Lambiase A (2018). Diagnosis and management of neuromyelitis optica spectrum disorders - An update. Autoimmun Rev.

[3] Tillema JM, Pirko I (2013). Neuroradiological evaluation of demyelinating disease. Ther Adv Neurol Disord.

[4] Trebst C, Jarius S, Berthele A, Paul F, Schippling S, Wildemann B (2014). Update on the diagnosis and treatment of neuromyelitis optica: Recommendations of the Neuromyelitis Optica Study Group (NEMOS).. J Neurol.

[5] Kim SM, Kim SJ, Lee HJ, Kuroda H, Palace J, Fujihara K (2017). Differential diagnosis of neuromyelitis optica spectrum disorders. Ther Adv Neurol Disord.

[6] Kawachi I, Lassmann H (2017). Neurodegeneration in multiple sclerosis and neuromyelitis optica. J Neurol Neurosurg Psychiatry.

[7] Kim GH, Kim JE, Rhie SJ, Yoon S (2015). The role of oxidative stress in neurodegenerative diseases. Exp Neurobiol.

[8] Nobrega-Pereira S, Fernandez-Marcos PJ, Brioche T, Gomez-Cabrera MC, Salvador-Pascual A, Flores JM (2016). G6PD protects from oxidative damage and improves healthspan in mice. Nat Commun.

[9] Tiwari M (2017). Glucose 6 phosphatase dehydrogenase (G6PD) and neurodegenerative disorders: Mapping diagnostic and therapeutic opportunities. Genes Dis.

[10] Siesjo BK (1978). Brain energy metabolism and catecholaminergic activity in hypoxia, hypercapnia and ischemia. J Neural Transm Suppl.

[11] Maker HS, Clarke DD, Lajtha A, Siegel GJ, Albers RW, Katzman R, Agranoff BW (1976). Intermediary metabolism of carbohydrates and amino acids. Basic neurochemistry.

[12] Bao BY, Ting HJ, Hsu JW, Lee YF (2008). Protective role of 1 alpha, 25-dihydroxyvitamin D3 against oxidative stress in nonmalignant human prostate epithelial cells. Int J Cancer.

[13] Chatterjee M (2001). Vitamin D and genomic stability. Mutat Res.

[14] Sardar S, Chakraborty A, Chatterjee M (1996). Comparative effectiveness of vitamin D3 and dietary vitamin E on peroxidation of lipids and enzymes of the hepatic antioxidant system in Sprague--Dawley rats. Int J Vitam Nutr Res.

[15] Hoeck AD, Pall ML (2011). Will vitamin D supplementation ameliorate diseases characterized by chronic inflammation and fatigue?. Med Hypotheses.

[16] Polman CH, Reingold SC, Banwell B, Clanet M, Cohen JA, Filippi M (2011). Diagnostic criteria for multiple sclerosis: 2010 revisions to the McDonald criteria. Ann Neurol.

[17] Wingerchuk DM, Lennon VA, Pittock SJ, Lucchinetti CF, Weinshenker BG (2006). Revised diagnostic criteria for neuromyelitis optica. Neurology.

[18] Mirshafiey A, Mohsenzadegan M (2009). Antioxidant therapy in multiple sclerosis. Immunopharmacol Immunotoxicol.

[19] Fischer MT, Sharma R, Lim JL, Haider L, Frischer JM, Drexhage J (2012). NADPH oxidase expression in active multiple sclerosis lesions in relation to oxidative tissue damage and mitochondrial injury. Brain.

[20] Calabrese V, Lodi R, Tonon C, D'Agata V, Sapienza M, Scapagnini G (2005). Oxidative stress, mitochondrial dysfunction and cellular stress response in Friedreich's ataxia. J Neurol Sci.

[21] Penton-Rol G, Cervantes-Llanos M, Martinez-Sanchez G, Cabrera-Gomez JA, Valenzuela-Silva CM, Ramirez-Nunez O (2009). TNF-alpha and IL-10 downregulation and marked oxidative stress in Neuromyelitis Optica. J Inflamm (Lond).

[22] Peng F, Yang Y, Liu J, Jiang Y, Zhu C, Deng X (2012). Low antioxidant status of serum uric acid, bilirubin and albumin in patients with neuromyelitis optica. Eur J Neurol.

[23] Balazs L, Leon M (1994). Evidence of an oxidative challenge in the Alzheimer's brain. Neurochem Res.

[24] Mejias R, Villadiego J, Pintado CO, Vime PJ, Gao L, Toledo-Aral JJ (2006). Neuroprotection by transgenic expression of glucose-6-phosphate dehydrogenase in dopaminergic nigrostriatal neurons of mice. J Neurosci.

[25] Garcion E, Wion-Barbot N, Montero-Menei CN, Berger F, Wion D (2002). New clues about vitamin D functions in the nervous system. Trends Endocrinol Metab.

